# KIF11 is required for proliferation and self-renewal of docetaxel resistant triple negative breast cancer cells

**DOI:** 10.18632/oncotarget.20785

**Published:** 2017-09-08

**Authors:** Meng Jiang, Huiru Zhuang, Rui Xia, Lei Gan, Yuantao Wu, Junzhe Ma, Yihui Sun, Zhixiang Zhuang

**Affiliations:** ^1^ Department of Oncology, The Second Affiliated Hospital of Soochow University, Suzhou, 215004, China; ^2^ Institute of Radiotherapy and Oncology, Soochow University, Suzhou, 215004, China; ^3^ Department of Plastic Surgery, The Second Affiliated Hospital of Soochow University, Suzhou, 215004, China; ^4^ Department of General Surgery, The Second Affiliated Hospital of Soochow University, Suzhou, 215004, China

**Keywords:** triple negative breast cancer, cancer stem cell, docetaxel resistance, kinesin family member 11

## Abstract

Development of chemoresistance remains a major hurdle for triple negative breast cancer treatment. Previous studies suggest that CD44+/CD24- cells, subpopulation of cancer stem cells with self-renewing and tumor-initiating capacities, are partly responsible for chemoresistance and therapeutic failure of triple negative breast cancer. Therefore, novel agents that target cancer stem cells (CSCs) may improve the clinical outcome. KIF11 (kinesin family member 11), overexpressed in many cancer cells, is a molecular motor protein that plays essential role in mitosis. In this study, we assess its role in docetaxel resistant triple negative breast cancer (TNBC). We found that the expression of KIF11 was significantly increased in CD44+/CD24- subpopulation of docetaxel resistant TNBC cells. Knockdown of KIF11 resulted in a significant decrease in the percentage of CSCs and mammosphere formation. KIF11 knockdown also inhibits cell growth and induces cell cycle G2/M arrest followed by cell mitosis and apoptosis. Further docetaxel resistant TNBC xenograft models demonstrated that KIF11 inhibitor exerts growth inhibitory effect *in vivo*. Of note, we also found that KIF11 was highly expressed in TNBC and its expression was correlated with shorter disease free survival time. All these data indicate that KIF11 is critical for proliferation and self-renewal in TNBC tumor cells *in vitro* and *in vivo*, suggesting that KIF11 may be a promising therapeutic target for treating chemoresistant TNBC.

## INTRODUCTION

Breast cancer is a highly heterogeneous malignancy that has become one of the leading causes of cancer-related death for women worldwide [1]. Triple negative breast cancer (TNBC), characterized by the lack expression of estrogen receptor (ER), progesterone receptor (PR), and human epidermal growth factor receptor 2 (HER2), accounts for approximately 15 to 20% of all breast cancers. Given that TNBC is not sensitive to endocrine therapy and HER2 targeted therapy, cytotoxic chemotherapy is the mainstay treatment [2, 3]. Docetaxel, which polymerizes tubulin to disrupt normal microtubule dynamics leading to cell death, is one of the most common chemotherapies for TNBC. Despite its initial efficacy in most patients with TNBC, the majority of these patients will relapse characterized by rapidly proliferating, drug-resistance and association with a high mortality rate [4]. Chemoresistance remains a major hurdle for the docetaxel based chemotherapy.

Analysis of human tumor tissues has provided evidence of a small population of stem-like cells that have been labeled as cancer stem cells (CSC). This population is able to dictate invasion, metastasis, and therapeutic resistance in tumors. Furthermore, it retains the capacity to self-renew and to differentiate [5, 6]. Breast cancer stem-like cell (BCSC) populations have been identified based on the cell membrane marker CD44+/CD24- [7]. TNBC with a higher percentage of CD44+/CD24− phenotypes were highly tumorigenic comparing to those with low percentage of CD44+/CD24− phenotypes [8, 9]. The discovery of CD44+/CD24- cells has generated excitement and led to efforts in multiple laboratories to find vulnerability for this subpopulation, as this subpopulation of cancer cells may represent a therapeutic target to anticancer drugs [10–12]. Besides, under certain circumstances, the non-CSC subpopulation can assume CSC properties. Thus, molecules or agents that could target both populations would therefore be expected.

KIF11 (kinesin family member 11), also known as kinesin-5, is a molecular motor protein that is essential in mitosis. It mediates centrosome separation and formation of the bipolar mitotic spindle, driving mitosis in order to support cell proliferation [13–16]. Inactivation of KIF11 results in inappropriate cell division and cell cycle arrest during mitosis, eventually leading to apoptotic cell death. KIF11 also appears to have non-mitotic functions as well [17]. It has also been proved to regulate axonal branching and growth cone motility and, more recently, shown to be involved in cell motility [18–20]. KIF11 has been demonstrated to over-express in various malignancies and correlated with their prognosis [21–23]. The critical role of KIF11 in mitotic progression as well as in cell motility makes it an attractive candidate for developing targeted therapy in cancer. We therefore sought to determine whether KIF11 is indeed an essential driver of both the proliferation and self-renewal of docetaxel resistant TNBC in this study.

## RESULTS

### Relationship between KIF11 expression and the stem cell-like characteristics in docetaxel resistant TNBC cells

**Figure 1:**
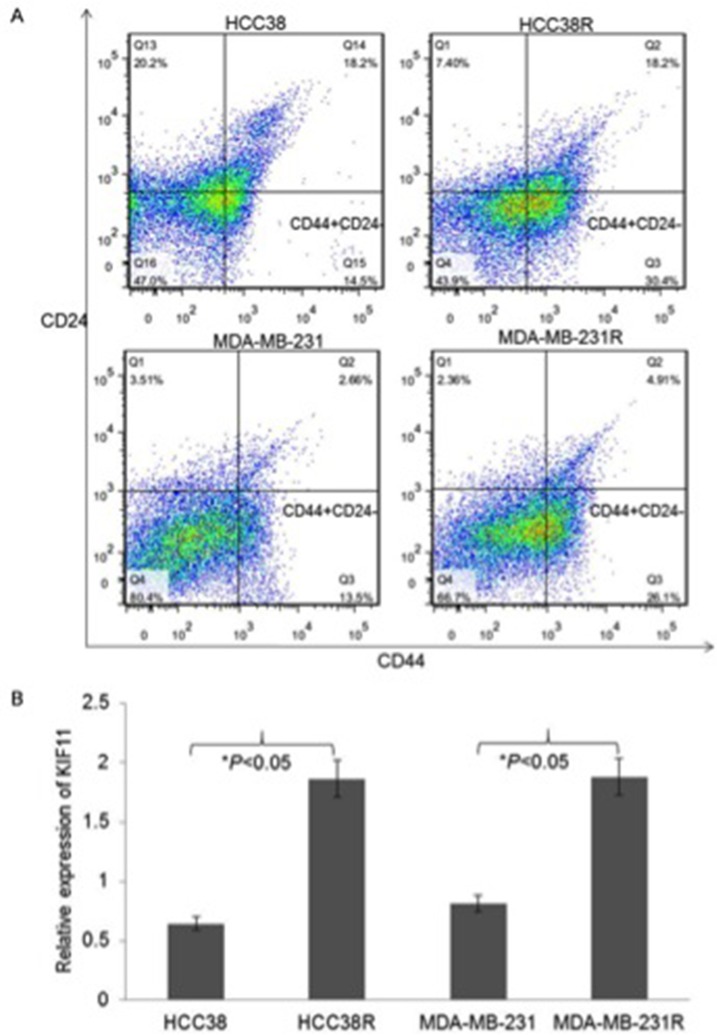
The stem-like and high expression of mitotic kinesin KIF11 cells were enriched in docetaxel resistant TNBC cells **(A)** Percentage of the different CD44+/CD24− subpopulations among HCC38, HCC38R, MDA-MB-231 and MDA-MB-231R cells was graphed and quantified using flow cytometry. **(B)** The mRNA expression levels of KIF11 in HCC38, HCC38R, MDA-MB-231 and MDA-MB-231R cells were measured by qRT-PCR. The mRNA levels of GAPDH were used as an internal control. The expression level of KIF11 was determined using 2 ^−ΔΔCt^ method.

Given that CD44+/CD24- cells in TNBC have been shown to possess tumor-initiating properties and to be associated with resistance to chemotherapy, we analyzed the two surface markers in both docetaxel sensitive and resistant TNBC cell lines. Consistent with the previous study, CD44+/CD24- phenotype percentage was significantly higher in the docetaxel resistant TNBC cells than their paternal TNBC cells (Figure 1A). We next analyzed the expression of KIF11 in the docetaxel resistant TNBC cell lines and their paternal cell lines in order to investigate whether KIF11 was involved in TNBC docetaxel resistance. According to the qRT-PCR analysis results, KIF11 mRNA level was significantly increased in both HCC38R and MDA-MB-231R cells (P<0.05, Figure 1B).

**Figure 2:**
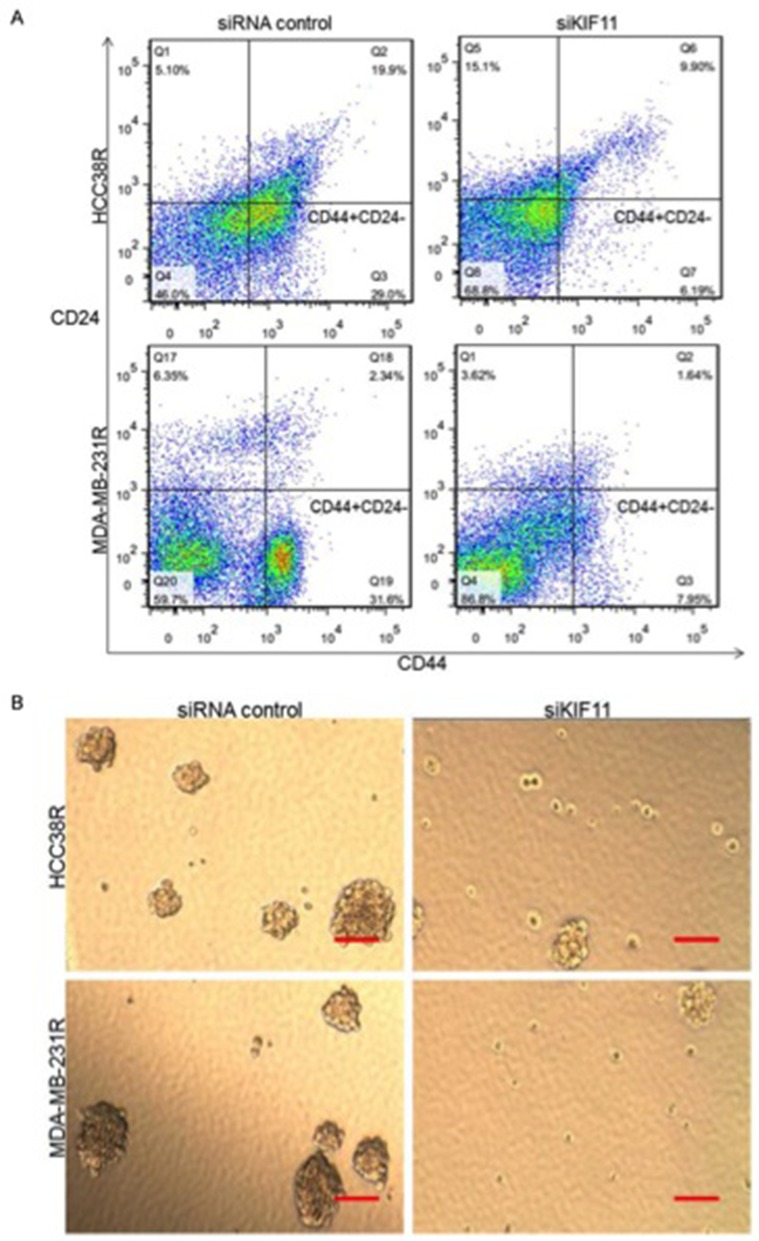
KIF11 is critical for self-renewal and stemness of docetaxel resistant TNBC cells **(A)** Percentage of CD44+/CD24− cells among the resistant TNBC cells after transfection with either si-control or si-KIF11 were measured via flow cytometry. **(B)** Representative images of mammosphere formation ability after transfection with either si-control or si-KIF11.

To further identify the relationship between KIF11 expression and the stem cell-like characteristics in docetaxel resistant TNBC cells, the effects of the KIF11 gene knockdown on the percentage of CD44+/CD24- cells were examined in HCC38R and MDA-MB-231R cells. As shown in Figure 2A, inhibition of KIF11 resulted in a significant decrease in the percentage of CD44+/CD24- cells in both cell lines. These results suggest that the KIF11 expression contributes to increasing of the CSC population. One of the hallmarks of the CSC is the formation of mammosphere in suspension cultures. To determine whether the decreasing expression of KIF11 reflected loss of self-renewal and stemness, we examined the mammosphere formation efficiency of HCC38R and MDA-MB-231R cells. Accordingly, although the mammosphere formation was observed after transfection with siKIF11, there was a dramatic reduction in the number and percentage of mammospheres compared to siRNA control group (Figure 2B). These results suggest that KIF11 decreasing could inhibit the growth of CD44+/CD24- cells in docetaxel resistant TNBC cells and abrogate the ability of self-renewing.

### Decreasing expression of KIF11 inhibits cell growth and induces cell cycle G2/M arrest in resistant TNBC cells

**Figure 3:**
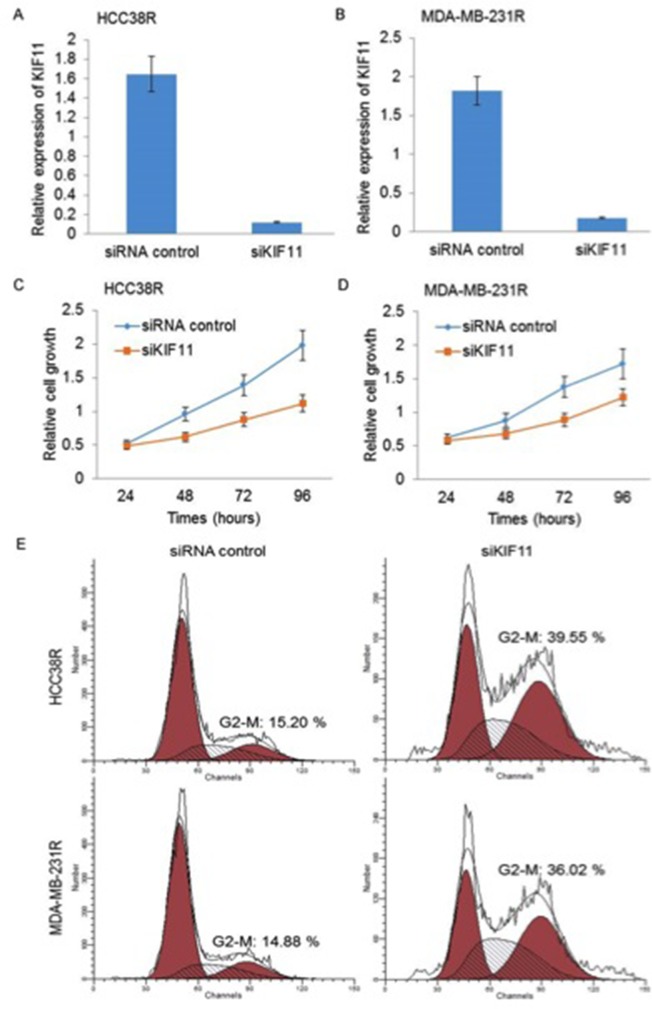
Silencing of KIF11 inhibits cell growth and induces cell cycle G2/M arrest in resistant TNBC cells **(A** and **B)** KIF11 mRNA levels were compared between si-control group and si-KIF11 group in HCC38R and MDR-MB-231R cells using qRT-PCR. **(C** and **D)** Relative cell growth at indicated time was compared between si-control group and si-KIF11 group in HCC38R and MDR-MB-231R cells by CCK8 assay. **(E)** Cell cycle after transfection was analyzed by flow cytometry.

As KIF11 has been considered to play an important role in proliferation and mitotic cycle, we were interested in studying the effect of decreasing expression of KIF11 on cell proliferation and cell cycle. After transfection, KIF11 expressions in HCC38R (Figure 3A) and MDA-MB-231R cells (Figure 3B) were examined by qRT-PCR. Based on the results of CCK8 assays, decreasing expression of KIF11 exhibited a potent inhibitory effect on the proliferation of docetaxel resistant TNBC cells compared to the control group (P<0.05, Figure 3C, 3D). We further investigated the effect of KIF11 decreasing on the cell cycle in docetaxel resistant TNBC cell lines using PI/RNase Staining Buffer detection kit. Cell cycle analysis demonstrated that decreasing expression of KIF11 resulted in a concomitant accumulation of cells in the G2/M phase (P<0.05, Figure 3E).

### Decreasing expression of KIF11 promotes docetaxel resistant TNBC cell mitosis and apoptosis

**Figure 4:**
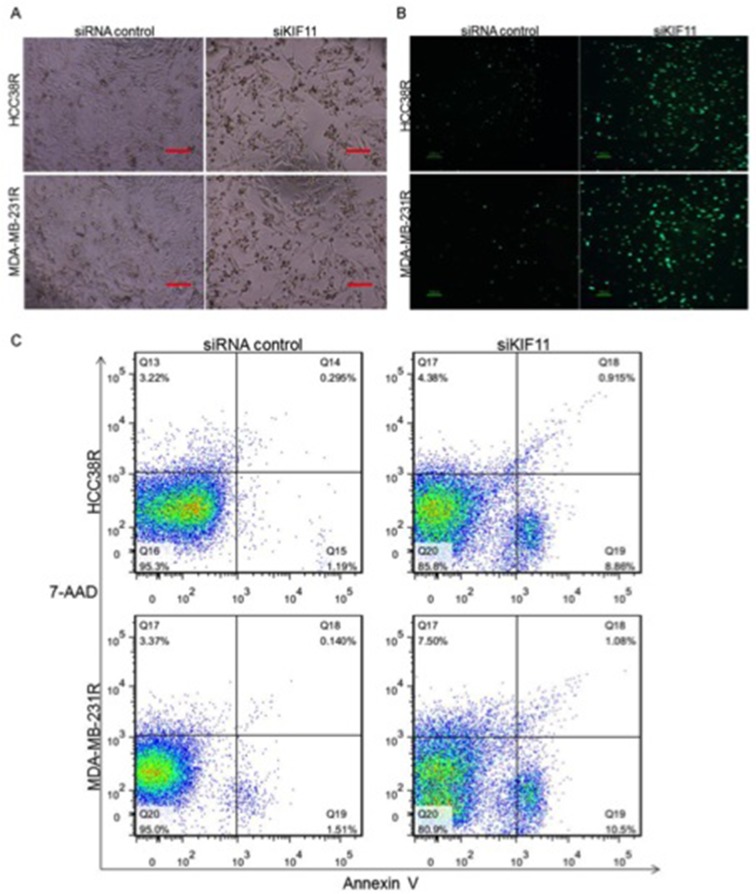
Silencing of KIF11 promotes docetaxel resistant TNBC cell mitosis and apoptosis **(A)** The morphology of docetaxel TNBC resistant cells after transfection with si-KIF11. **(B)** H3-P expression in HCC38R and MDA-MB-231R cells after transection with siRNA control or siKIF11. **(C)** Apoptosis induced by KIF11 knockdown was analyzed by Annexin V–FITC labeling.

Our results so far show that decreasing expression of KIF11 inhibits cell growth and induces cell cycle G2/M arrest, and we therefore need to further clarify its effect on cell mitosis and apoptosis in resistant TNBC cells to assess whether KIF11 may be a very compelling therapeutic target. Figure 4A showed significant morphological alterations of both HCC38R and MDA-MB-231R cells after transfection with si-KIF11, such as reduction in cell size and minor cell detachment. We also observed a significant reduction of cell number after transfection with si-KIF11 groups in comparison to the control groups.

High levels of cell-cycle-dependent phosphorylation of histone H3 (H3-P), which takes place in most eukaryotes, constitute a conserved hallmark of mitotic cell division [24]. We therefore assessed the cell mitosis in resistant TNBC cells by H3-P immunostaining. The results showed that level of H3-P in cells transfected with si-KIF11 were significantly higher than their control groups (Figure 4B), which suggest that decreasing expression of KIF11 promotes docetaxel resistant TNBC cell mitosis. The following Annexin V/7-AAD assay provided evidence that decreasing expression of KIF11 also induced apoptosis of resistant TNBC cells (P<0.05, Figure 4C).

### Decreasing expression of KIF11 inhibits migration and invasion of docetaxel resistant TNBC cells

**Figure 5:**
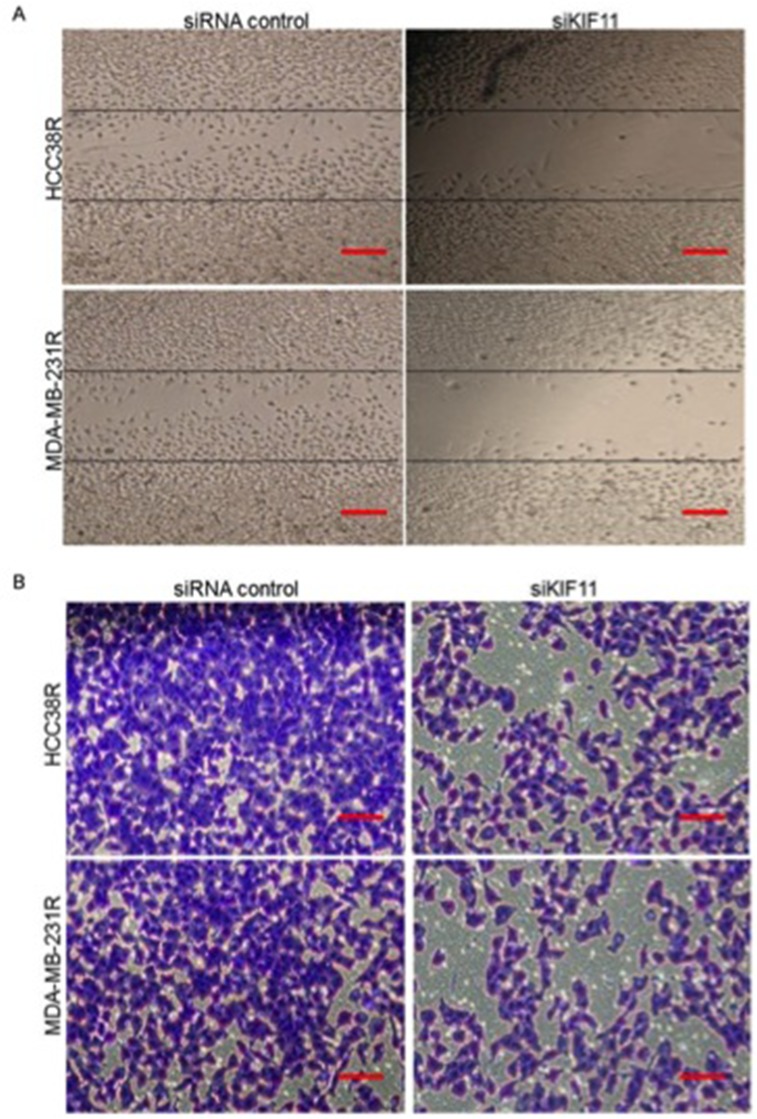
Knockdown of KIF11 inhibits migration and invasion of resistant TNBC cells **(A)** Scratch wound assays were performed in HCC38R and MDA-MB-231R cells transfected with siRNA control or siKIF11. Images were taken at 0 and 24 hours after confluent monolayers of cells were wounded. **(B)** Chambers invasion assays were performed to evaluate the invasion ability of resistant TNBC cells transfected with siRNA control or siKIF11.

Docetaxel resistant TNBC tumor cells often possess enhanced migration and invasion capabilities. As KIF11 was also shown to be involved in cell motility, we next sought to determine the effect of KIF11 inhibition on these capabilities of resistant TNBC cells. The effect of KIF11 inhibition on cell migration was examined using an established scratch assay. Cells transfected with si-control were much efficient in migrating and approximately covering the wounded area, while the si-KIF11 groups nearly completely lost their migratory ability (Figure 5A). The vitro invasion assay showed that inhibition of KIF11 inhibited the capacity of invasion (Figure 5B). All of these results showed that decreasing expression of KIF11 inhibits migration and invasion of docetaxel resistant TNBC cells.

### KIF11 inhibitor induces tumor shrinkage in docetaxel resistant TNBC xenograft models

**Figure 6:**
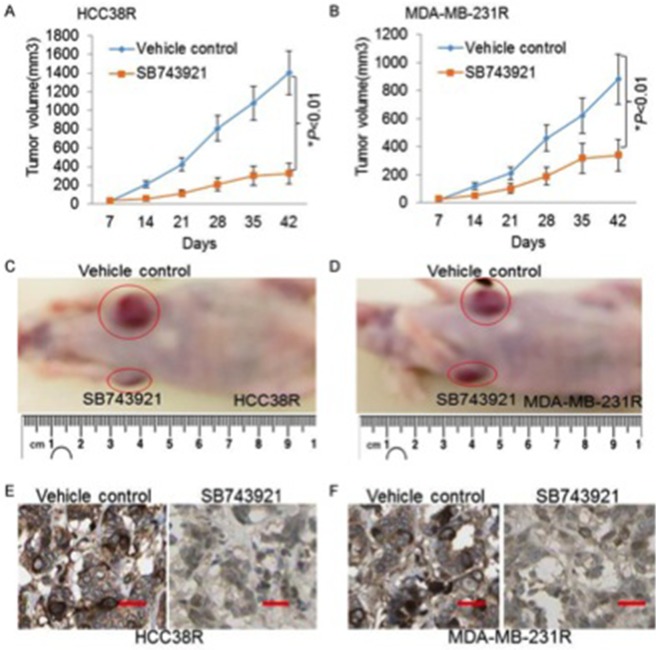
The KIF11 inhibitor induces significantly tumor regression in the docetaxel resistant TNBC xenograft models **(A** and **B)** Progression of tumor volumes during days 7–42 treated with vehicle or SB743921 in the HCC38R and MDA-MB-231R xenograft models. **(C** and **D)** Images showing the tumor received vehicle control or SB743921 treatment in the HCC38R and MDA-MB-231R xenograft models. **(E** and **F)** Representative images of KIF11 expression analyzed by immunohistochemical staining in tumor specimens are shown.

Docetaxel resistant TNBC xenograft models were established to evaluate the efficacy of KIF11 inhibition on tumor progression *in vivo*. After intratumoral injection treatment for 6 times, the mean tumor volumes were 1,299±238 mm^3^ and 1,034±178 mm^3^ for the vehicle control groups of HCC38R and MDA-MB-231R, respectively; whereas the mean tumor volumes for the SB743921 groups were 219±141 mm^3^ and 234±178 mm^3^, respectively (P<0.05, Figure 6A, 6B). It was apparent that tumor growth was effectively controlled by KIF11 inhibitor SB743921 (Figure 6C). Immunohistochemistry staining was used to assess the expression of KIF11 on tumor tissues, as shown in Figure 6D. As for the expression of KIF11 in SB743921 treated groups compared to the control groups, positive cells significantly decreased. Altogether, these data indicated that KIF11 could be a therapeutic target in treating resistant TNBC, as KIF11 inhibitor significantly inhibits the tumor growth *in vivo*.

### KIF11 was increased in TNBC and associated with patients’ survival

**Figure 7:**
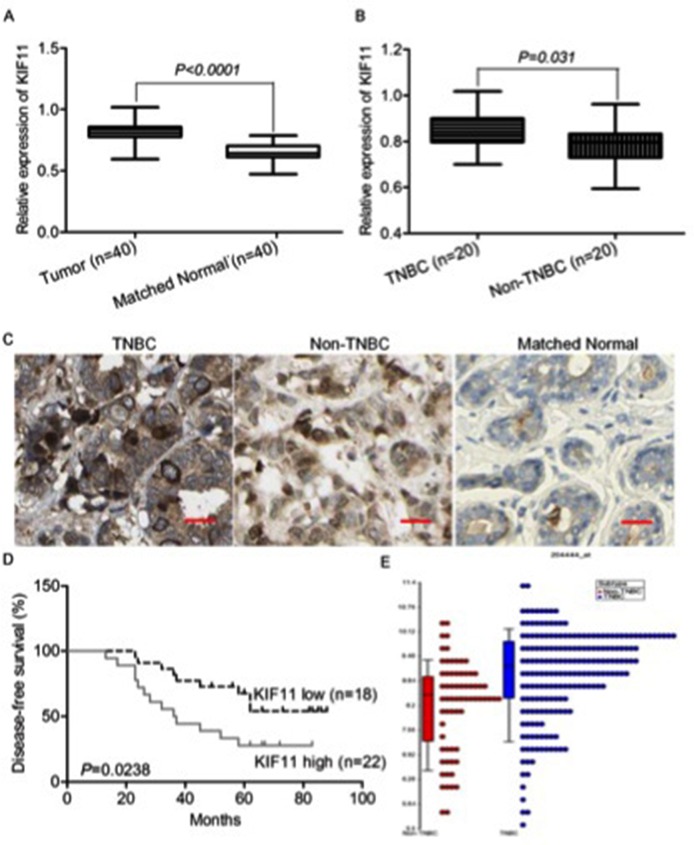
KIF11 is up-regulated in TNBC and associated with patients’ survival **(A)** qRT-PCR analysis of KIF11 gene status in 40 TNBC tissues compared with 40 matched normal tissues. **(B)** qRT-PCR analysis of KIF11 gene status in 20 TNBC tissues compared with 20 non-TNBC tissues. **(C)** Representative images of KIF11 immunohistochemical staining in TNBC, non-TNBC and matched normal tissues are shown. **(D)** Kaplan–Meier curves for disease-free survival (DFS) were compared according to different expression of KIF11 to assess its prognostic significance in TNBC patients (n = 40). **(E)** Dot plot for comparison of KIF11expression between TNBC and non-TNBC patients in TCGA database.

To further explore the clinical significance of KIF11 in the prognosis of TNBC, we examined the KIF11 expression in 20 non-TNBC, 20 TNBC samples and 40 matched normal tissues of patients. As shown in Figure 7A, KIF11 mRNA was significantly higher in the tumor samples resected from the breast cancer patients and much lower in the matched normal tissues (P<0.001). When compared to the non-TNBC tissues, the KIF11 mRNA expression was still higher (Figure 7B, P=0.031). The results of KIF11 immunohistochemical staining were in consistent with the previous results (Figure 7C). Subsequently, we divided 40 TNBC specimens into high- and low- KIF11 expression groups according to the median value. We found that the disease free survival time of the low KIF11 expression group was significantly longer than that of the high expression group (Figure 7D, P=0.0238). We further analyzed KIF11 expression levels across different breast cancer subtypes in TCGA (The Cancer Genome Atlas) database, the results revealed that high KIF-11 expression correlated with the more aggressive TNBC subtype, compared to non-TNBC tumors (Figure 7E).

## DISCUSSION

As a subset of breast cancer presenting highly aggressive clinical behaviors and poor prognosis, TNBC is becoming the focus of recent research and clinical studies. To date, combination of conventional cytotoxic chemotherapeutic agents remains the standard therapy because of the fact that no single targeted therapy has been significantly effective for TNBC tumors. But targeted therapy options for TNBC treatment have also been studied in clinical trials. Cetuximab, an anti-EGFR antibody, plus carboplatin treatment demonstrated responses in fewer than 20% of metastatic TNBC patients according to a randomized phase II trial [25]. AZD2281, a PARP inhibitor, resulted in progression free survival of approximately six months in patients with BRCA1- or BRCA2-deficient advanced breast cancer (of which 50% were triple-negative) [26]. Therefore, understanding the molecular mechanisms for development of chemoresistance in TNBC and identification of attractive molecule targets is of significant importance for chemoresistant TNBC treatment [27].

Tubulin is a heterodimeric protein that plays an intrinsic role in cytoskeleton and mitotic spindle physiology [28]. Agents targeting microtubule such as docetaxel belong to the most successful anticancer agents. Unfortunately, despite its significant anticancer efficacy, many patients develop resistance to these agents eventually. The mitotic kinesin KIF11, expressed and active only during mitosis, plays a vital role in mitotic progression. Overexpression of KIF11 was proved to cause mitotic defects leading to genome instability and tumorigenesis in transgenic mice [29]. The discovery of small-molecule KIF11 inhibitors spurred strong interest in their development as anticancer agents and some of them have entered clinical trials [30, 31]. Studies have demonstrated that inhibition of KIF11 could induce apoptosis and overcome drug resistance in various cancer cells [32–34]. In consideration of the promising results reported by others, we aim to investigate the role KIF11 in resistant TNBC cells.

CSCs has been reported to not only initiate and sustain tumor growth, but also play a major role in tumor metastasis and resistance to current chemotherapeutic agents [35]. Thus, therapeutic strategies targeting and eliminating CSCs have emerged to treat TNBCs. Breast CSCs was found displaying a profile of cell surface markers characterized by the phenotype CD44+/CD24-. Consistent with previous reports, we observed that the docetaxel resistant TNBC cells was enriched with the stem-like cell population with increased expression of mitotic kinesin KIF11 compared to their parental doceteaxel sensitive cells. Results of our study demonstrated that inhibition of KIF11 caused a significant decrease in the percentage of CD44+/CD24- cells and repression of mammosphere formation in HCC38R and MDA-MB-231R cells. Mammosphere formation is considered to be an assay for evaluating the stemness of cells and it requires proliferation as well as self-renewal activity of cells. These results suggest that the KIF11 expression contributes to the self-renewal and stemness in docetaxel resistant cell lines.

Cell proliferation, invasion and metastasis are crucial processes related to tumorigenesis and tumor progression. In our study, KIF11 knockdown inhibits cell proliferation and induces cell cycle G2/M arrest followed by cell mitosis and apoptosis. KIF11 may also play an important role in the invasion and metastasis of resistant TNBC cells, as it was shown to be involved in cell motility [20]. Indeed, we found decreased migration and invasion ability after inhibition of KIF11. Besides, the *in vivo* growth inhibitory effect of KIF11 inhibitor is being analyzed with docetaxel resistant TNBC xenograft models. As expected, KIF11 inhibitor significantly reduced the tumor volume.

Clinical samples were collected to verify the hypothesis that the expression of KIF11 is associated with the prognosis of the patients with TNBC. KIF11 is reported highly expressed in proliferating compared with non-proliferating cancer cells [36]. Data of our study showed that KIF11 expression was significantly increased in primary tumors from TNBC patients as compared to matched normal tissues and non-TNBC tumors, in consistent with previous reports. Additionally, increasing of KIF11 in TNBC was proved to be associated with inferior disease free survival, indicating that elevated levels of KIF11 have a vital role in TNBC prognosis. And the KIF11 expression comparison between TNBC and non-TNBC demonstrates that high level of KIF11 expression tends to be a more aggressive tumor subtype.

In conclusion, in this study, we present extensive evidence to demonstrate that KIF11 is critical for proliferation and self-renewal in TNBC tumor cells, *in vitro* and *in vivo*. These findings suggest that KIF11 may represent a promising molecule target for treating docetaxel resistant TNBC.

## MATERIALS AND METHODS

### Cell culture

The human TNBC cell lines HCC38 and MDA-MB-231 were obtained from Cell Bank of Type Culture Collection of Chinese Academy of Sciences (Shanghai, China). Cells were cultured in RPMI 1640 medium (GIBCO, Grand Island, NY, USA) supplemented with 10% fetal bovine serum (GIBCO), 1% penicillin/streptomycin at 37°C in a humidified 5% CO_2_ atmosphere. Docetaxel resistant cell lines (HCC38R and MDA-MB-231R) were established by stepwise selection with an increasing concentration of docetaxel, preserved by our lab.

### Clinical specimens

All TNBC samples, non-TNBC tissues and matched normal tissues were obtained from patients at the Department of General Surgery, the Second Affiliated Hospital to Soochow University, China, between January 2014 and January 2016. The fresh tissue samples were immediately immersed in RNAlater (Qiagen, Germany) after surgical resection, stored at 4°C overnight and subsequently frozen in liquid nitrogen for storage at -80°C until analysis. The tissue samples were collected and used after obtaining approval from the Ethics Committee of the Second Affiliated Hospital to Soochow University. Written informed consent was obtained from all of the patients who participated in this study according to committee's regulations. Disease free survival was defined as the interval between date of diagnosis and first recurrence or death. The prognostic effect of KIF11 was evaluated using the Kaplan–Meier method and compared using the log-rank test.

### Flow cytometry

For staining of CD44 and CD24, cells were exposed to trypsin, washed and suspended in PBS containing pre-conjugated primary antibodies: CD24-PE (1:20, eBioscience, USA); CD44-FITC (1:50, eBioscience) and incubated with the antibodies for 30 min at 4°C. Unstained cells were used for negative control. Cells that only stained with CD24-PE were used to regulate compensation and set CD44-FITC gate, while Cells that only stained with CD44-FITC were used to regulate compensation and set CD24-PE gate. The labeled cells were washed, fixed, and then analyzed with a FACSCalibur Flow Cytometry (BD, USA).

Cells cycle arrest rate was detected by flow cytometry using Cell Cycle Detection Kit (Key-GEN, Nanjing, China). Following its manufacturer's instructions, 2 mL suspension of 10^6^ cells was fixed with 70% ethyl alcohol, and washed by PBS before being stained. The rate of each cycle was analyzed by FACSCalibur Flow Cytometry at 488 nm.

Cells apoptotic rate was also detected by flow cytometry using FITC Annexin V Apoptosis Detection Kit with 7-AAD (Key-GEN, Nanjing, China) according to the manufacturer's instructions. Two mL suspension of 10^5^ cells was stained with (Annexin-V-FITC and 7-AAD) kit solution in dark for 15 min. The apoptosis rate was assayed using FACSCalibur Flow Cytometry at 488 nm.

### Quantitative real-time PCR

Total RNA was extracted using TRIZOL (Takara) and then reverse-transcribed to cDNA using PrimeScript Reverse Transcriptase (Takara). Quantitative real-time PCR (qRT-PCR) was performed using the ABI prism 7300-sequence detection system (Applied Biosystems, USA). The following cycle parameters were used: denaturation at 95°C for 30 s followed by annealing for 30s at 58°C, and elongation for 30s at 72°C. The following primers were used: acacttgtgagaactgaacc (sense), cacggctcttgacttacg (anti-sense) for KIF11, and ccttcattgacctcaactacatg (sense), cttctccatggtggtgaaga (anti-sense) for GADPH. The relative mRNA expression levels were calculated by the comparative Ct method, normalized with the average expression of GAPDH.

### Transient transfection

For siRNA transfection, experimental conditions were optimized. siRNA experiments were carried out in serum- and antibiotic-free RPMI1640 medium (GIBCO) using RNAi MAX transfect agent (Invitrogen). Cells were plated overnight at 50% to 70% confluence and then transfected with either siRNA control or KIF-specific siRNAs (QIAGEN). Cells were lysed or tested in functional assays 24h or 48 h after transfection. Transfection efficiency was evaluated by qRT-PCR.

### Mammosphere assay

Single cell suspensions were seeded in 6-well ultra-low attachment plates (Corning Life Sciences, USA). Every three days, fresh mammosphere media was added. Mammosphere number and volume were determined using the GelCount™ mammalian cell colony counter (Oxford Optronix). For microscopic images of mammospheres the original magnification of the images are 40x. Each experiment was done in triplicates and repeated three times.

### Cell proliferation assay

Cell proliferation was measured using the CCK8 method. In brief, cells were seeded onto 96-well plates at a density of 5*×*10^3^ cells/well 24 h after transfection and cultured for up to another 72 h. Cell Counting Kit-8 (CCK-8, Dojindo) was added into each well according to the manufacturer's instructions and incubated for 2 h every 24h to measure cell proliferation. Then the optical density (OD) at 490 nm was measured with a microplate reader (Molecular Devices Corp., Sunnyvale, USA) from three independent experiments to calculate the mean and standard error, and considered to be directly proportional to the number of living cells in the culture.

### Immunostaining

Cells were seeded onto 12 mm round glass coverslips placed in the base of each well of a 24-well plate. After adhesion, cells were transfected. 48 h after the transfection, cells were washed with PBS, fixed with 3.7% formaldehyde, permeabilized with 0.1% Triton X-100 and blocked in PBS supplemented with 1% skimmed milk, 2.5% bovine serum albumin (BSA) and 8% fetal bovine serum (FBS) at room temperature. Cells were incubated overnight with human anti-H3-P-FITC antibody (1:500, eBioscience) at 4°C for 30 minutes. The expression of H3-P was observed by fluorescence microscopy.

### Wound healing assay

Cells were seeded in 12 well plates until they reached 60–70% confluency after transfection for 48h. After 24 h, cell monolayer was scraped in a straight line to create a “scratch” with a p200 pipet tip. The cells were washed with growth medium to remove debris and to smooth the edge of the scratch, then replaced with 1 mL growth medium and incubated for 24 h. Images were taken at 0 and 24 hour by placing the 12 well plate under phase contrast microscope (Olympus IX81 automated Inverted) using automated Stage-Pro program to ensure the same area is aligned and photographed.

### Cell invasion assay

Growth factor-reduced BD matrigel™ invasion chambers (24-well plate, 8.0μm, BD BioCoat™) were used (BD Biosciences, San Jose, CA, USA). Invasive cells were Crystal Violet stained 7 days later and photographed. Each experiment was done in triplicates and repeated three times.

### Docetaxel resistant TNBC xenograft models

Female Nu/Nu mice (4 to 6 weeks old) were injected with 2 × 10^6^ of HCC38R or MDA-MB-231R cells in the second left and second right mammary gland. Seven days after the injection of tumor cells, every mouse were to receive vehicle treatment in the second left mammary gland and KIF11 inhibitor SB743921 treatment in the second right mammary gland by intratumoral injection every seven days for six times per experiment. Tumor volume was measured by caliper. At the end point, tumors were collected, fixed in10% formalin and immunohistochemically analyzed.

### Immunohistochemistry procedure

UltraSensitive S-P IHC Kit (Maixin, Fuzhou, China) was used for immunohistochemical staining according to the manufacturer`s protocols. The sections were incubated with anti-KIF11 antibody (1:100, Abcam, USA) at 4°C overnight. Then they were stained by a streptavidin-peroxidase system and the signal was visualized using diaminobenzidine substrate.

### Statistical analysis

The results were presented as mean ± standard deviation from triplicate repeated three separate times and analyzed with SPSS software (version 22.0; SPSS Inc, Chicago, IL, USA) unless otherwise indicated. The significance of differences was determined by one-way analysis of variance among multiple groups, and P<0.05 was considered statistically significant.

## References

[1] Assi HA, Khoury KE, Dbouk H, Khalil LE, Mouhieddine TH, El Saghir NS (2013). Epidemiology and prognosis of breast cancer in young women. J Thorac Dis.

[2] Liedtke C, Mazouni C, Hess KR, Andre F, Tordai A, Mejia JA, Symmans WF, Gonzalez-Angulo AM, Hennessy B, Green M, Cristofanilli M, Hortobagyi GN, Pusztai L (2008). Response to neoadjuvant therapy and long-term survival in patients with triple-negative breast cancer. J Clin Oncol.

[3] Brouckaert O, Wildiers H, Floris G, Neven P (2012). Update on triple-negative breast cancer: prognosis and management strategies. Int J Womens Health.

[4] Carey LA, Dees EC, Sawyer L, Gatti L, Moore DT, Collichio F, Ollila DW, Sartor CI, Graham ML, Perou CM (2007). The triple negative paradox: primary tumor chemosensitivity of breast cancer subtypes. Clin Cancer Res.

[5] Visvader JE, Lindeman GJ (2012). Cancer stem cells: current status and evolving complexities. Cell Stem Cell.

[6] Reya T, Morrison SJ, Clarke MF, Weissman IL (2001). Stem cells, cancer, and cancer stem cells. Nature.

[7] Al-Hajj M, Wicha MS, Benito-Hernandez A, Morrison SJ, Clarke MF (2003). Prospective identification of tumorigenic breast cancer cells. Proc Natl Acad Sci U S A.

[8] Idowu MO, Kmieciak M, Dumur C, Burton RS, Grimes MM, Powers CN, Manjili MH (2012). CD44(+)/CD24(-/low) cancer stem/progenitor cells are more abundant in triple-negative invasive breast carcinoma phenotype and are associated with poor outcome. Hum Pathol.

[9] Giatromanolaki A, Sivridis E, Fiska A, Koukourakis MI (2011). The CD44+/CD24- phenotype relates to ‘triple-negative’ state and unfavorable prognosis in breast cancer patients. Med Oncol.

[10] Pece S, Tosoni D, Confalonieri S, Mazzarol G, Vecchi M, Ronzoni S, Bernard L, Viale G, Pelicci PG, Di Fiore PP (2010). Biological and molecular heterogeneity of breast cancers correlates with their cancer stem cell content. Cell.

[11] Ma F, Li H, Wang H, Shi X, Fan Y, Ding X, Lin C, Zhan Q, Qian H, Xu B (2014). Enriched CD44(+)/CD24(-) population drives the aggressive phenotypes presented in triple-negative breast cancer (TNBC). Cancer Lett.

[12] Al-Hajj M, Becker MW, Wicha M, Weissman I, Clarke MF (2004). Therapeutic implications of cancer stem cells. Curr Opin Genet Dev.

[13] Mayer TU, Kapoor TM, Haggarty SJ, King RW, Schreiber SL, Mitchison TJ (1999). Small molecule inhibitor of mitotic spindle bipolarity identified in a phenotype-based screen. Science.

[14] Sarli V, Giannis A (2008). Targeting the kinesin spindle protein: basic principles and clinical implications. Clin Cancer Res.

[15] Kahn OI, Sharma V, Gonzalez-Billault C, Baas PW (2015). Effects of kinesin-5 inhibition on dendritic architecture and microtubule organization. Mol Biol Cell.

[16] Sawin KE, LeGuellec K, Philippe M, Mitchison TJ (1992). Mitotic spindle organization by a plus-end-directed microtubule motor. Nature.

[17] Uzbekov R, Prigent C, Arlot-Bonnemains Y (1999). Cell cycle analysis and synchronization of the Xenopus laevis XL2 cell line: study of the kinesin related protein XlEg5. Microsc Res Tech.

[18] Falnikar A, Tole S, Baas PW (2011). Kinesin-5, a mitotic microtubule-associated motor protein, modulates neuronal migration. Mol Biol Cell.

[19] Wang F, Lin SL (2014). Knockdown of kinesin KIF11 abrogates directed migration in response to epidermal growth factor-mediated chemotaxis. Biochem Biophys Res Commun.

[20] Ferhat L, Cook C, Chauviere M, Harper M, Kress M, Lyons GE, Baas PW (1998). Expression of the mitotic motor protein Eg5 in postmitotic neurons: implications for neuronal development. J Neurosci.

[21] Ding S, Xing N, Lu J, Zhang H, Nishizawa K, Liu S, Yuan X, Qin Y, Liu Y, Ogawa O, Nishiyama H (2011). Overexpression of Eg5 predicts unfavorable prognosis in non-muscle invasive bladder urothelial carcinoma. Int J Urol.

[22] Liu L, Liu X, Mare M, Dumont AS, Zhang H, Yan D, Xiong Z (2016). Overexpression of Eg5 correlates with high grade astrocytic neoplasm. J Neurooncol.

[23] Liu M, Wang X, Yang Y, Li D, Ren H, Zhu Q, Chen Q, Han S, Hao J, Zhou J (2010). Ectopic expression of the microtubule-dependent motor protein Eg5 promotes pancreatic tumourigenesis. J Pathol.

[24] Escriba MC, Goday C (2013). Histone H3 phosphorylation and elimination of paternal X chromosomes at early cleavages in sciarid flies. J Cell Sci.

[25] Carey LA, Rugo HS, Marcom PK, Mayer EL, Esteva FJ, Ma CX, Liu MC, Storniolo AM, Rimawi MF, Forero-Torres A, Wolff AC, Hobday TJ, Ivanova A (2012). TBCRC 001: randomized phase II study of cetuximab in combination with carboplatin in stage IV triple-negative breast cancer. J Clin Oncol.

[26] Audeh MW, Carmichael J, Penson RT, Friedlander M, Powell B, Bell-McGuinn KM, Scott C, Weitzel JN, Oaknin A, Loman N, Lu K, Schmutzler RK, Matulonis U (2010). Oral poly(ADP-ribose) polymerase inhibitor olaparib in patients with BRCA1 or BRCA2 mutations and recurrent ovarian cancer: a proof-of-concept trial. Lancet.

[27] Yadav S, Sehrawat A, Eroglu Z, Somlo G, Hickey R, Yadav S, Liu X, Awasthi YC, Awasthi S (2013). Role of SMC1 in overcoming drug resistance in triple negative breast cancer. PLoS One.

[28] Wiltshire C, Singh BL, Stockley J, Fleming J, Doyle B, Barnetson R, Robson CN, Kozielski F, Leung HY (2010). Docetaxel-Resistant Prostate Cancer Cells Remain Sensitive to S-Trityl-L-Cysteine-Mediated Eg5 Inhibition. Molecular Cancer Therapeutics.

[29] Castillo A, Morse HC, Godfrey VL, Naeem R, Justice MJ (2007). Overexpression of Eg5 causes genomic instability and tumor formation in mice. Cancer Res.

[30] Rath O, Kozielski F (2012). Kinesins and cancer. Nat Rev Cancer.

[31] Owens B (2013). Kinesin inhibitor marches toward first-in-class pivotal trial. Nat Med.

[32] Yin Y, Sun H, Xu J, Xiao F, Wang H, Yang Y, Ren H, Wu CT, Gao C, Wang L (2015). Kinesin spindle protein inhibitor SB743921 induces mitotic arrest and apoptosis and overcomes imatinib resistance of chronic myeloid leukemia cells. Leuk Lymphoma.

[33] Carter BZ, Mak DH, Shi Y, Schober WD, Wang RY, Konopleva M, Koller E, Dean NM, Andreeff M (2006). Regulation and targeting of Eg5, a mitotic motor protein in blast crisis CML: overcoming imatinib resistance. Cell Cycle.

[34] Sun L, Lu J, Niu Z, Ding K, Bi D, Liu S, Li J, Wu F, Zhang H, Zhao Z, Ding S (2015). A Potent Chemotherapeutic Strategy with Eg5 Inhibitor against Gemcitabine Resistant Bladder Cancer. PLoS One.

[35] Wicha MS, Liu S, Dontu G (2006). Cancer stem cells: an old idea--a paradigm shift. Cancer Res.

[36] Piao XM, Byun YJ, Jeong P, Ha YS, Yoo ES, Yun SJ, Kim WJ (2016). Kinesin Family Member 11 mRNA Expression Predicts Prostate Cancer Aggressiveness. Clin Genitourin Cancer.

